# Real-Life Retrospective Turkiye Data of the De-Escalation of ABVD to AVD in Hodgkin Lymphoma: On Behalf of the TSH Turkish Lymphoma Study Group

**DOI:** 10.3390/jcm14196813

**Published:** 2025-09-26

**Authors:** Emel Isleyen, Nurcan Alhan, Esra Terzi Demirsoy, Ayfer Geduk, Duygu Nurdan Avci, Mahmut Yeral, Ahmet Burhan Ferhanoglu, Ebru Pekguc, Eren Gunduz, Hava Uskudar Teke, Nihan Alkis, Zafer Serenli Yegen, Fahir Ozkalemkas, Tuba Ersal, Volkan Karakus, Fatma Aykas, Irfan Yavasoglu, Ayse Hilal Eroglu Kucukdiler, Ozan Salim, Gulsum Ozet, Simten Dagdas, Sule Mine Ozturk, Merve Ecem Erdogan Yon, Ozge Soyer Kosemehmetoglu, Emine Merve Savas, Seyma Yildiz, Selami Kocak Toprak, Muhit Ozcan, Guldane Cengiz Seval, Leylagul Kaynar, Sureyya Yigit Kaya, Erman Ozturk, Pinar Tiglioglu, Ahmet Kursat Gunes, Selin Kucukyurt, Cem Selim, Tayfur Toptas, Meral Ulukoylu Menguc, Fatma Arikan, Fatma Keklik Karadag, Hale Bulbul, Aysun Senturk Yikilmaz, Ekin Kircali, Selin Merih Urlu, Deniz Goren, Elif Birtas Atesoglu, Omur Gokmen Sevindik, Fatos Dilan Koseoglu, Taha Ulutan Kars, Atakan Tekinalp, Serkan Guven, Ozgur Mehtap

**Affiliations:** 1 Ankara Bilkent City Hospital Hematology Clinic, Ankara 06800, Türkiye; dremel.isleyen@gmail.com (E.I.); gulsumozet@gmail.com (G.O.); simtendagdas@gmail.com (S.D.); sulemine.ozturk@yahoo.com (S.M.O.); merveecemerdogan@hotmail.com (M.E.E.Y.); doozge@hotmail.com (O.S.K.); 2 Education and Research Hospital Hematology Clinic, Alanya Alaaddin Keykubat University, Antalya 07425, Türkiye; nurcanalhan@gmail.com; 3 Faculty of Medicine Hematology Clinic, Kocaeli University, Kocaeli 41380, Türkiye; esraterzi@gmail.com (E.T.D.); ayfergeduk@hotmail.com (A.G.); 4 Adana Dr. Turgut Noyan Practice and Research Center Hematology Clinic, Başkent University, Adana 01250, Türkiye; nurduav@hotmail.com (D.N.A.); drmyeral@gmail.com (M.Y.); 5 Hematology Clinic, American Hospital, Istanbul 34365, Türkiye; bferhan@gmail.com (A.B.F.); ebrupekguc@gmail.com (E.P.); 6 Faculty of Medicine, Hematology Clinic, Eskişehir Osmangazi University, Eskisehir 26480, Türkiye; erengunduz26@gmail.com (E.G.); havaus@yahoo.com (H.U.T.); 7 Hematology Clinic, Bursa City Hospital, Bursa 16250, Türkiye; nihanalkis@hotmail.com; 8 Hematology Clinic, Private Doruk Nilüfer Hospital, Bursa 16110, Türkiye; zaferserenli83@yahoo.com; 9 Hematology Clinic, Bursa Uludağ University, Bursa 16059, Türkiye; fahir@uludag.edu.tr (F.O.); tubaersal@uludag.edu.tr (T.E.); 10 Hematology Clinic, Antalya Education and Research Hospital, Antalya 07100, Türkiye; dr_v_karakus@yahoo.com (V.K.); fatmaaykas@hotmail.com (F.A.); 11 Hematology Clinic, Aydın Adnan Menderes University, Aydın 09100, Türkiye; dryavas09@gmail.com (I.Y.); e_hilal@hotmail.com (A.H.E.K.); 12 Faculty of Medicine, Hematology Clinic, Akdeniz University, Antalya 07059, Türkiye; ozansalim@gmail.com; 13 Faculty of Medicine, Hematology Clinic, Gazi University, Ankara 06500, Türkiye; drmervesavas@gmail.com (E.M.S.); yildizseyma2014@hotmail.com (S.Y.); 14 Faculty of Medicine, Hematology Clinic, Ankara University, Ankara 06590, Türkiye; sktoprak@yahoo.com (S.K.T.); muhit1907@gmail.com (M.O.); guldanecengiz@gmail.com (G.C.S.); 15 Hematology Clinic, Medipol University, Istanbul 34214, Türkiye; drleylagul@gmail.com (L.K.); sureyyayigitkaya@hotmail.com (S.Y.K.); 16 Department of Hematology, Faculty of Medicine, Istanbul Medeniyet University, Istanbul 34720, Türkiye; ermanozturk@gmail.com (E.O.); dr.pinarakyol@hotmail.com (P.T.); 17 Hematology Clinic, Ankara Etlik City Hospital, Ankara 06170, Türkiye; ahmetkgunes@gmail.com (A.K.G.); dr.skucukyurt@hotmail.com (S.K.); 18 Adult Hematology Department, Konya Selçuk University, Konya 42250, Türkiye; dr.cemselim@gmail.com; 19 Faculty of Medicine, Hematology Clinic, Marmara University, Istanbul 34899, Türkiye; toptast@gmail.com (T.T.); u_meral@hotmail.com (M.U.M.); fatmagecgelarikan@gmail.com (F.A.); 20 Hematology Clinic, Izmir Tepecik Training and Research Hospital, Izmir 35020, Türkiye; fatma_keklik86@hotmail.com (F.K.K.); halebulbul95@yahoo.com (H.B.); 21 Hematology Clinic, Denizli State Hospital, Denizli 20010, Türkiye; senturkaysun@gmail.com; 22 Hematology Clinic, Medicana International Ankara Hospital, Ankara 06510, Türkiye; ekinkircali@gmail.com (E.K.); smerihoz@gmail.com (S.M.U.); 23 Hematology Clinic, Istanbul Florence Nightingale Hospital, Istanbul 34381, Türkiye; drdenizgoren@gmail.com; 24 Hematology Clinic, Yeditepe University Hospital, Istanbul 34752, Türkiye; ebirtas@yahoo.com; 25 Faculty of Medicine, Demiroğlu Science University, Istanbul 34394, Türkiye; omurgok17@hotmail.com; 26 Hematology Clinic, Çiğli Training and Research Hospital, Izmir Bakırçay University, Izmir 35620, Türkiye; fatosdilankoseoglu@gmail.com; 27 Hematology Clinic, Konya City Hospital, Konya 42020, Türkiye; tahaulutankars@gmail.com; 28 Faculty of Medicine, Hematology Clinic, Necmettin Erbakan University, Konya 42080, Türkiye; atakantekinalp@hotmail.com; 29 Hematology Clinic, Çanakkale Mehmet Akif Ersoy State Hospital, Çanakkale 17000, Türkiye; drserkanguven@gmail.com

**Keywords:** Hodgkin’s lymphoma, ABVD, bleomycin omission, de-escalation, PET-CT

## Abstract

**Background:** Classical Hodgkin lymphoma (cHL) demonstrates high survival rates with the ABVD regimen (doxorubicin, bleomycin, vinblastine, dacarbazine); however, the use of bleomycin is associated with a significant risk of pulmonary toxicity. The Risk-Adapted Treatment of HL (RATHL) trial demonstrated that omitting bleomycin in patients with favorable interim Positron Emission Tomography (PET-CT) results did not adversely affect survival outcomes. In this study, we present real-world data from advanced-stage HL patients treated according to the RATHL protocol. **Methods:** This multicenter, retrospective study included newly diagnosed cHL patients with Ann Arbor stage IIB–IV disease or stage IIA disease with bulky disease or with involvement of three or more sites, enrolled from 29 centers across Türkiye. The analysis focused on patients whose initial treatment was de-escalated from ABVD to AVD (bleomycin was omitted). Data were collected on demographic and clinical prognostic characteristics, interim PET-CT findings (evaluated using the Deauville score), progression-free survival (PFS) and overall survival (OS). Survival outcomes were assessed using Kaplan–Meier analysis. **Results:** A total of 379 patients were included, with a median age of 34 years (range: 18–78). Following interim PET-CT assessments (After 2 cycles of ABVD), Deauville scores were 1 in 39.8% of patients, 2 in 39.1%, and 3 in 21.1%. Based on these results, bleomycin was omitted immediately after interim PET-CT in 73.9% of patients, after one additional ABVD cycle in 12.1%, and after two additional cycles in 14%. The median follow-up duration was 28 months (range: 6–96). The 3-year PFS and OS rates were 86.0% and 96.1%, respectively. Patients with Deauville scores of 1–2 had a 3-year PFS rate of 87.6%, compared to 79.8% in those with a score of 3 (*p* = 0.087). Increased age, poor Eastern Cooperative Oncology Group Scale (ECOG) performance status, bulky disease, and higher International Prognostic Scores (IPS) were significantly associated with inferior OS (*p* < 0.05). There were no significant differences in OS among patients who received 2, 3, or 4 cycles of ABVD. However, among patients treated with 2 cycles of ABVD, both extranodal involvement (*p* = 0.039) and higher IPS (*p* = 0.002) were significantly associated with decreased PFS. **Conclusions:** Our findings demonstrate that PET-guided de-escalation of bleomycin after two cycles of ABVD is feasible, effective, and safe in real-world multicenter practice in Türkiye. The survival outcomes are comparable to those reported in the RATHL study, reinforcing the role of interim PET-CT in guiding individualized therapy. However, patients with high IPS or extranodal involvement may require more tailored management strategies.

## 1. Introduction

Current treatment approaches for classical Hodgkin lymphoma (cHL) yield 5-year OS (overall survival) rates of approximately 92% for early-stage (Stage I–II) disease and 82% for advanced-stage (Stage III–IV) disease [1].

In patients with advanced-stage disease or early-stage disease accompanied by adverse prognostic features, intensive chemotherapy regimens such as ABVD (doxorubicin, bleomycin, vinblastine, dacarbazine) or BEACOPP (bleomycin, etoposide, doxorubicin, cyclophosphamide, vincristine, procarbazine, and prednisone) or BrECADD [2] (Brentuximab vedotoin, etoposide, doxorubicin, cyclophosphamide dacarbazine, dexametazone) or Nivolumab + AVD or Brentuksimab + AVD [3] are widely used and have demonstrated efficacy. With these new approaches, PFS (progression-free survival) and OS (overall survival) have improved. In many developing countries, the ABVD regimen remains the standard of care for cHL, primarily due to the cost-prohibitive nature of novel therapeutic agents [4].

However, a key objective in contemporary research is to identify prognostic markers that can guide de-escalation of therapy in selected patients, thereby reducing long-term treatment-related toxicities without compromising efficacy.

Several critical questions remain unresolved in the management of advanced-stage HL. These include whether current prognostic tools are adequate for identifying patients who truly require intensive first-line therapy, and whether patients who undergo treatment de-escalation—thus avoiding long-term adverse effects of chemotherapy—achieve outcomes comparable to those who receive full-intensity regimens.

The RATHL trial (The Risk-Adapted Treatment of HL) provided pivotal evidence that in patients with advanced-stage or high-risk early-stage HL, negative interim PET (Positron Emission Tomography) scans can guide safe de-escalation from ABVD to AVD (i.e., omission of bleomycin), significantly reducing the risk of long-term toxicity [5]). This strategy not only preserves treatment efficacy but also enhances quality of life and minimizes unnecessary exposure to potentially harmful agents. In contrast, a positive interim PET scan indicates residual disease activity and necessitates escalation to more intensive treatment.

In the present study, we aimed to present real-world data reflecting outcomes of HL patients in Türkiye who were treated according to the RATHL protocol—data not previously reported in the literature. Specifically, we sought to highlight the prognostic utility of interim PET imaging in informing treatment de-escalation decisions in patients with advanced-stage or high-risk early-stage HL, and to report survival outcomes in this cohort.

## 2. Methods

This retrospective, cross-sectional study was conducted by the Turkish Lymphoma Study Group under the auspices of the Lymphoma Scientific Subcommittee of the Turkish Society of Hematology (TSH). A total of 29 centers across Türkiye participated in the study. **Eligibility criteria** included adult patients diagnosed with classical Hodgkin lymphoma (cHL) at stage IIB–IV disease or stage IIA disease with bulky disease or with involvement of three or more sites who received ABVD as initial therapy and subsequently underwent treatment de-escalation from ABVD to AVD (i.e., omission of bleomycin) at any point during their treatment course. Patients with incomplete or missing data were excluded. Post-interim-PET decisions (ABVD → AVD de-escalation versus administration of 1–2 additional ABVD cycles before bleomycin omission) were made at physician discretion and were not dictated by a uniform study algorithm. Across centers, the intended approach was RATHL-concordant de-escalation for Deauville 1–3; however, additional cycles were sometimes given based on baseline disease features (e.g., bulky disease (>33% of the transthoracic diameter or >10 cm elsewhere)), operational considerations (e.g., delayed PET reporting), and overall clinical judgment. Because this was a retrospective study, the *specific documented reason* for extending ABVD was inconsistently recorded and was therefore not analyzed as a prespecified variable. Bleomycin toxicity in patients was evaluated by pulmonary function tests, computed tomography, and measurement of carbon monoxide diffusion capacity.

Clinical data were collected for patients treated between December 2014 and May 2024. Data were extracted from the electronic hospital information system, encompassing demographic distribution, baseline characteristics, clinical parameters and PET-CT findings. Interim PET responses, whether classified as partial or complete metabolic, were verified using the available reports. The data collection was finalized in December 2024, at which time all surviving patients were censored. The primary endpoint of the study was to determine whether the progression-free survival (PFS) and overall survival (OS) outcomes of all patients were consistent with those reported in the de-escalation arm of the RATHL trial. The secondary endpoints were to evaluate PFS and OS differences across subgroups and to assess the extent to which real-world clinical practice strictly adhered to the RATHL protocol.

## 3. Statistical Analysis

Statistical analyses were conducted using IBM^®^ SPSS Statistics version 27. Descriptive statistics are presented as frequencies with percentages or as medians accompanied by ranges (minimum–maximum). Categorical variables were compared using the Chi-square test, whereas the Mann–Whitney U test was employed to assess differences between two independent groups for non-parametric data. PFS was defined as the interval from diagnosis to the first documented disease progression or death from any cause, whichever occurred first. OS was calculated from the date of diagnosis to death from any cause. Survival outcomes were analyzed using the Kaplan–Meier method and differences across prognostic subgroups were evaluated using the log-rank test. Three- year survival rates are reported as percentages with corresponding standard errors (SEs). A two-sided *p*-value of <0.05 was considered statistically significant.

## 4. Results

### 4.1. Patient Characteristics

A total of 379 patients (50.4% male) with a median age of 34 years (range: 18–78) were included in the study. The baseline clinical characteristics are summarized in Table 1. Following two cycles of ABVD chemotherapy, interim PET-CT assessments revealed a Deauville score of 1–2 in 302 patients (79.7%) and a score of 3 in 77 patients (20.3%). No significant differences were observed between patients with a Deauville score of 1–2 and those with a score of 3 regarding age at diagnosis, gender, ECOG performance status, Ann Arbor stage, presence of B symptoms and presence of bulky disease or extranodal involvement (Table 1). However, Deauville score 3 was significantly more frequently observed in 30 of 112 patients with an International Prognostic Score (IPS) ≥ 3 (26.8%), compared to 47 of 267 patients with an IPS of 0–2 (17.6%) (*p* = 0.043).

In the cohort, de-escalation of therapy by omission of bleomycin was implemented immediately after the interim PET-CT in 280 patients (73.9%). An additional cycle or two of ABVD was administered before bleomycin discontinuation in 46 (12.1%) and 53 (14.0%) patients, respectively. Patients who received additional ABVD before omission more often had bulky disease (2 cycles: 16.1% vs. 3 cycles: 34.8% vs. 4 cycles: 34.0%; *p* < 0.001). In contrast, interim PET response was not worse in these groups (Deauville 3: 22.9% vs. 13.0% vs. 13.2%, respectively), suggesting that decisions to continue ABVD were influenced by baseline risk and logistical factors rather than PET status alone. None of the patients received consolidation radiotherapy.

Bleomycin-induced pulmonary toxicity was documented in six patients (1.5%). Among these six patients, three received four cycles of ABVD, one received three cycles, and two received two cycles. In all cases, symptom onset occurred after completion of therapy. Grade I–II pulmonary toxicity was documented in all patients. Four patients achieved complete resolution with corticosteroid therapy, whereas the treatment modality and clinical outcomes of the remaining two patients were not available.

A comparison of the clinical characteristics of patients who received 2, 3, or 4 cycles of ABVD prior to bleomycin omission is presented in Table 2.

### 4.2. Survival Analyses

During a median follow-up period of 28 months (range: 6–96 months), 40 patients (10.6%) experienced disease relapse. Of these, 27 had undergone treatment de-escalation to AVD after two cycles of ABVD, 8 had received one additional cycle of bleomycin, and 5 had received two additional cycles. A total of 10 patients (2.6%) died during follow-up. Among them, 7 had de-escalated to AVD after two cycles, 2 had received one additional cycle, and 1 had received two additional cycles of bleomycin.

The median PFS and overall survival OS durations were not reached. The estimated 3-year PFS and OS rates were 86.0% (standard error [SE]: 2.1) and 96.1% (SE: 1.2), respectively.

No significant associations with PFS were observed for age at diagnosis (<45 vs. ≥45 years; *p* = 0.801), sex (*p* = 0.492), ECOG performance status (0–1 vs. 2–3; *p* = 0.106), Ann Arbor stage (*p* = 0.155), presence of B symptoms (*p* = 0.242), bulky disease (*p* = 0.720) or extranodal involvement (*p* = 0.128). However, a high IPS (IPS ≥ 3 vs. 0–2) was significantly associated with inferior PFS (*p* = 0.001).

**Figure 1:**
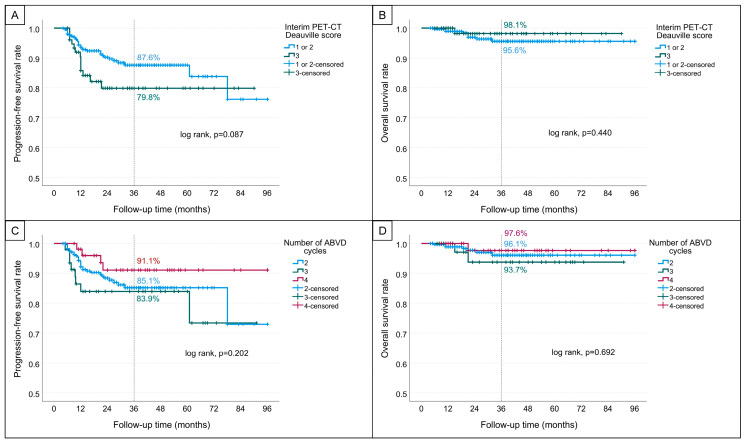
Kaplan–Meier survival curves. (**A**,**B**) show progression-free survival and overall survival, respectively, stratified by interim PET-CT Deauville scores. (**C**,**D**) show progression-free survival and overall survival, respectively, according to the number of ABVD chemotherapy cycles received.

Based on interim PET-CT findings, the 3-year PFS was 87.6% (SE: 2.3) in patients with a Deauville score of 1–2, and 79.8% (SE: 5.1) in those with a score of 3 (*p* = 0.087; Figure 1A). According to the number of ABVD cycles received before bleomycin omission, the 3-year PFS rates were 85.1% (SE: 2.6) in those who received 2 cycles, 83.9% (SE: 5.6) in those who received 3 cycles, and 91.1% (SE: 4.3) in those who received 4 cycles (*p* = 0.202; Figure 1C).

Factors significantly associated with inferior OS included older age at diagnosis (<45 vs. ≥45 years; *p* = 0.007), poor ECOG performance status (0–1 vs. 2–3; *p* < 0.001), presence of bulky disease (*p* = 0.019) and higher IPS ( ≥ 3 vs. 0–2; *p* = 0.003). Sex (*p* = 0.493), Ann Arbor stage (*p* = 0.054), B symptoms (*p* = 0.090) and interim PET-CT Deauville score (*p* = 0.440) were not significantly associated with OS (Figure 1B, Table 3).

The 3-year OS rates stratified by the number of ABVD cycles received were 96.1% (SE: 1.5) for 2 cycles, 93.7% (SE: 4.3) for 3 cycles and 97.6% (SE: 2.4) for 4 cycles (*p* = 0.692; Figure 1D).

### 4.3. Patients De-Escalated After 2 Cycles of ABVD

Among the 280 patients (50.4% female) who received two cycles of ABVD followed by de-escalation, the median age at diagnosis was 34 years (range: 18–78). The median follow-up duration was 28 months (range: 6–96 months). In this subgroup, the estimated 3-year PFS and OS rates were 85.1% and 96.1%, respectively. Patients with an interim PET-CT Deauville score of 1–2 had a 3-year PFS rate of 86.9%, whereas those with a score of 3 had a PFS rate of 79.1% (*p* = 0.102).

Baseline clinical and demographic characteristics for all subgroups are presented in Table 2. Within this subgroup, a Deauville score of 3 (versus 1–2) on interim PET-CT was significantly associated with male sex (*p* = 0.040; Table 4). However, other factors—including age at diagnosis, sex, ECOG performance status, Ann Arbor stage, presence of B symptoms, bulky disease and Deauville score—were not significantly associated with PFS.

Conversely, a high IPS (IPS ≥3 vs. 0–2; *p* = 0.002) and the presence of extranodal involvement (*p* = 0.039) were significantly associated with inferior PFS outcomes. Factors associated with reduced OS in this subgroup included older age at diagnosis (<45 vs. ≥45 years; *p* = 0.002), high IPS (≥3 vs. 0–2; *p* = 0.009), poor ECOG performance status (2–3 vs. 0–1; *p* = 0.002) and the presence of B symptoms (*p* = 0.042), as summarized in Table 5.

## 5. Discussion

In this study, we aimed to analyze the demographic characteristics and survival outcomes of newly diagnosed cHL patients in Türkiye who were de-escalated to AVD after achieving a Deauville score of 1–3 on interim PET following two cycles of ABVD. Our objective was to present real-world data reflecting the RATHL study context. The primary strength of real-world data lies in its inclusion of patients irrespective of restrictive clinical trial criteria, such as ECOG performance status or comorbidities.

One striking observation in our real-world data was that, although all centers performed interim PET after two cycles—similar to the RATHL study—some physicians postponed de-escalation by administering additional cycles of ABVD based on clinical judgment. In particular, several centers gave two further cycles of ABVD before switching to AVD in patients with negative interim PET findings. This approach appeared more common in patients with bulky disease, possibly reflecting concern about relapse, and was occasionally influenced by delayed PET reporting. Consequently, certain patients received three or four cycles of ABVD prior to bleomycin omission. This subgroup demonstrated a higher prevalence of bulky disease and a lower proportion of Deauville 3 responses compared with those de-escalated earlier. Importantly, survival outcomes did not differ according to the number of pre-omission cycles, reinforcing that extending bleomycin exposure did not provide measurable benefit while potentially increasing the risk of toxicity. While delayed PET reporting was noted anecdotally by some investigators and may have contributed to postponement of de-escalation, the exact number of patients affected could not be determined due to inconsistent documentation across centers. The principal limitation of our study, therefore, is the absence of systematic documentation regarding the specific reasons for extending ABVD beyond two cycles. As a result, we were unable to determine the exact proportion of patients who received additional cycles exclusively due to delayed PET reporting.

The demographic characteristics of our cohort of 280 patients who underwent de-escalation after two cycles of ABVD were largely comparable to those reported in the RATHL study [5]. The median age was 34 years (range: 18–78), with 49.6% of patients being male, whereas in the RATHL study, the median age was 33 years (range: 18–79) and 54.2% of patients were male. Disease stage distribution in our cohort included 37.1% with stage II, 31.8% with stage III and 31.1% with stage IV disease, which closely mirrors the RATHL study (Stage II: 42.4%, Stage III: 30.1%, Stage IV: 27.5%). B symptoms were present in 60.7% of our patients, similar to the 59.6% reported in the RATHL cohort. However, the incidence of bulky disease was lower in our population (16.1%) compared to the RATHL study (32.3%).

In the RATHL study, following a median follow-up of 41.2 months, the 3-year PFS and OS rates in the AVD arm were reported as 84.4% and 97.6%, respectively. At 7 years, the PFS and OS rates were 79.2% and 93.5%, respectively [6]. In our study, with a shorter median follow-up of 28 months, patients who underwent de-escalation after two cycles of ABVD demonstrated a PFS of 85.1% and an OS of 96.1%. Although these findings appear comparable to those of the RATHL study, the relatively limited follow-up in our cohort represents an important limitation, underscoring the need for longer observation to allow more robust comparisons with long-term trial outcomes. Moreover, patients who were de-escalated after receiving one or two additional doses of bleomycin exhibited similar survival outcomes to those de-escalated after two cycles of ABVD. During the early phase of treatment, bleomycin-related toxicity was observed in six of the 379 patients. Among these, two patients underwent de-escalation following two cycles of ABVD, whereas three of the remaining four patients were de-escalated after four cycles. Notably, the administration of additional bleomycin doses in iPET-negative patients did not confer any survival benefit. The presence of pulmonary toxicity in 6 of our patients supports, in line with the literature, that treatment de-escalation in interim PET-negative patients helps protect against bleomycin-related side effects. In the RATHL trial, pulmonary toxicity occurred in 7% of patients in the ABVD arm compared with 3% in the AVD arm, highlighting the clinical advantage of bleomycin omission [5]. In our study, the incidence of bleomycin-induced pulmonary toxicity was even lower at 1.5%, which may reflect the real-world practice of early de-escalation and supports the safety of limiting bleomycin exposure. Taken together, these findings emphasize the critical role of interim PET-guided treatment in personalizing therapy for Hodgkin lymphoma, as it not only maintains efficacy but also minimizes the risk of bleomycin-induced pulmonary toxicity. Among our six affected patients, four achieved complete resolution with corticosteroid therapy, whereas the treatment approach and outcomes of the remaining two could not be determined. Nevertheless, due to the retrospective nature of our analysis, detailed information regarding clinical presentation, diagnostic approaches, and management strategies of pulmonary toxicity was not systematically available and represents a limitation of our study.

Among patients who underwent de-escalation after two cycles of ABVD, similar to the RATHL study, the presence of B symptoms, bulky disease, interim PET status, and gender did not significantly impact PFS. However, higher IPS (*p* = 0.002) and the presence of extranodal disease (*p* = 0.039) were associated with worse PFS. On the other hand, when the analysis was extended to include all patients, the presence of extranodal involvement (*p* = 0.12) did not reach statistical significance, while high IPS remained a significant predictor of inferior PFS (*p* = 0.001). The absence of a significant PFS difference in the overall cohort may be attributed to the administration of one or two additional cycles of bleomycin, which could have mitigated the negative impact of extranodal disease. Nonetheless, the smaller sample size of our study compared to the RATHL trial necessitates further research with larger patient populations to validate these findings. When evaluated in terms of PFS, our study and the RATHL trial yielded comparable results with respect to the IPS; however, in the RATHL trial, patients with extranodal disease were not analyzed as separate subgroups. The baseline extranodal involvement rate for all participants in the RATHL study was not stated directly. Nevertheless, assessments based on initial PET imaging showed that 14% of patients had disease upstaging, with 74% (n:118) of these upstaging phenomena being ascribed to the discovery of extranodal illness [7]. As a result, it is possible to infer that approximately 10% of RATHL study participants had extranodal involvement at baseline. When compared to the real-world data in our study, this estimated rate appears to be considerably lower. In a real-world study evaluating 169 patients, 20 of the 101 iPET-negative patients were de-escalated to AVD, and in this subgroup the 3-year PFS rate was reported as 90%. Among patients who continued ABVD, the rate was 83.3%. Although based on smaller patient numbers, these results are consistent with our study [8].

Additionally, while the RATHL study reported worse PFS with increasing Ann Arbor stage and older age, we observed that PFS in our study was not affected by age or stage. This suggests potential differences between trial populations and real-world practice.

In conclusion, the interim PET-guided treatment approach enables the implementation of equally effective yet less toxic strategies in the treatment of Hodgkin lymphoma, improving patient treatment outcomes. One of the key contributions of our study lies in its demonstration that treatment individualization guided by interim PET-CT is feasible and broadly applicable across multicenter hematology practice in Türkiye. However, notable inter-institutional variations—such as the continuation of ABVD beyond interim PET despite negative images—resulted in deviations from standard protocols. This highlights the fact that, in real-world clinical practice, therapeutic decisions are often influenced by patient-specific factors and strict adherence to established treatment algorithms may not always be observed. Furthermore, the relatively inferior PFS observed in certain subgroups, particularly those with extranodal involvement and high IPS, underscores the need for further investigation through larger, more powered cohorts. Unlike the RATHL trial, which did not evaluate extranodal disease as a distinct prognostic variable, the present study offers a novel perspective by bringing this aspect into focus. Consequently, de-escalation decisions should be approached with greater caution in patients presenting with extranodal disease. Nonetheless, the relatively small size of certain subgroups and the lack of systematic documentation regarding reasons for extended ABVD highlight the need for larger, prospective studies to further refine risk-adapted strategies in daily practice. Overall, this study provides the first real-world evidence from Türkiye supporting the implementation of interim PET-guided therapy, marking a significant step toward the individualization of treatment strategies in Hodgkin lymphoma.

## 6. Conclusions

Ultimately our findings highlight the critical role of real-world data in bridging the gap between clinical trial evidence and daily practice in Hodgkin lymphoma. By identifying extranodal disease as a distinct subgroup with inferior PFS, our study offers novel insights that emphasize the need for greater caution in de-escalation decisions and may inform future risk-adapted therapeutic strategies.

## Figures and Tables

**Table 1:** Association between patient characteristics and treatment response on interim PET-CT.

Characteristics	Total (n = 379)	Interim PET-CT Deauville Score		
		Score 1 or 2 (n = 302)	Score 3 (n = 77)	*p* Value
Age median (range), yr	34 (18–78)	33 (18–78)	37 (18–73)	0.380
Age at diagnosis, n (%)				0.707
18–24 years	90 (23.7)	72 (23.8)	18 (23.4)	
25–44 years	168 (44.3)	137 (45.4)	31 (40.3)	
45–59 years	71 (18.7)	56 (18.5)	15 (19.5)	
≥60 years	50 (13.2)	37 (12.3)	13 (16.9)	
Male sex, n (%)	191 (50.4)	146 (48.3)	45 (58.4)	0.114
ECOG-PS, n (%)				0.179
0	199 (52.5)	164 (54.3)	35 (45.5)	
1	144 (38.0)	114 (37.7)	30 (39.0)	
2	32 (8.4)	21 (7.0)	11 (14.3)	
3	4 (1.1)	3 (1.0)	1 (1.3)	
Ann Arbor stage, n (%)				0.316
II	142 (37.5)	113 (37.4)	29 (37.7)	
III	117 (30.9)	98 (32.5)	19 (24.6)	
IV	120 (31.7)	91 (30.1)	29 (37.7)	
B symptoms, n (%)	237 (62.5)	185 (61.3)	52 (67.5)	0.310
Bulky disease, n (%)	79 (20.8)	61 (20.2)	18 (23.4)	0.540
Extranodal involvement, n (%)	112 (29.6)	86 (28.5)	26 (33.8)	0.364
International prognostic score, n (%)				**0.043**
0–2	267 (70.4)	220 (72.8)	47 (17.6)	
≥3	112 (29.6)	82 (27.2)	30 (26.8)	

Bold text indicates statistical significance at *p* < 0.05 level. *Abbreviations:* ECOG-PS: Eastern Cooperative Oncology Group-Performance Status, PET-CT: Positron Emission Tomography-Computed Tomography.

**Table 2:** Comparison of the characteristics of patients who underwent 2, 3, and 4 cycles of ABVD.

Characteristics	Number of ABVD Cycles			*p* Value
	2 (n = 280)	3 (n = 46)	4 (n = 53)	
Age at diagnosis, n (%)				0.456
18–44 years	188 (67.1)	35 (76.1)	35 (66.0)	
≥45 years	92 (32.9)	11 (23.9)	18 (34.0)	
Male sex, n (%)	139 (49.6)	22 (47.8)	30 (56.6)	0.606
ECOG-PS, n (%)				0.944
0 or 1	254 (90.7)	41 (89.1)	48 (90.6)	
2 or 3	26 (9.3)	5 (10.9)	5 (9.4)	
Ann Arbor stage, n (%)				0.776
II	104 (37.1)	15 (32.6)	23 (43.4)	
III	89 (31.8)	15 (32.6)	13 (24.5)	
IV	87 (31.1)	16 (34.8)	17 (32.1)	
B symptoms, n (%)	170 (60.7)	29 (63.0)	38 (71.7)	0.317
Bulky disease, n (%)	45 (16.1)	16 (34.8)	18 (34.0)	**<0.001**
Extranodal involvement, n (%)	84 (30.0)	9 (19.6)	19 (35.8)	0.198
International prognostic score, n (%)				0.315
0–2	201 (71.8)	28 (60.9)	38 (71.7)	
≥3	79 (28.2)	18 (39.1)	15 (28.3)	
Interim PET-CT Deauville score				0.118
1 or 2	216 (77.1)	40 (87.0)	46 (86.8)	
3	64 (22.9)	6 (13.0)	7 (13.2)	

Bold text indicates statistical significance at *p* < 0.05 level. *Abbreviations:* ABVD: Adriamycin (doxorubicin), Bleomycin, Vinblastine, and Dacarbazine, ECOG-PS: Eastern Cooperative Oncology Group-Performance Status, PET-CT: Positron Emission Tomography-Computed Tomography.

**Table 3:** Relationship between prognostic factors and progression-free survival and overall survival.

Characteristics	Groups	Progression-Free Survival			Overall Survival		
		Events/n	3-Year PFS, % (SE)	*p* Value	Events/n	3-Year OS, % (SE)	*p* Value
Age at diagnosis	18–44 yr	29/258	86.3 (2.4)	0.801	3/258	98.2 (1.0)	**0.007**
	≥45 yr	15/121	92.1 (2.5)		7/121	91.6 (3.2)	
Sex	Male	20/191	88.8 (2.6)	0.492	4/191	96.7 (1.7)	0.493
	Female	24/188	83.2 (3.3)		6/188	95.5 (1.8)	
ECOG-PS	0 or 1	37/343	87.1 (2.1)	0.106	5/343	97.7 (1.1)	**<0.001**
	2 or 3	7/36	76.7 (8.0)		5/36	81.8 (7.5)	
Ann Arbor stage	II	13/142	88.6 (3.1)	0.155	1/142	99.0 (1.0)	0.054
	III	12/117	89.1 (3.2)		3/117	96.5 (2.0)	
	IV	19/120	78.8 (4.9)		6/120	91.7 (3.5)	
B symptoms	Absent	12/142	88.6 (3.2)	0.242	1/142	98.8 (1.2)	0.090
	Present	32/237	84.6 (2.7)		9/237	94.6 (1.8)	
Bulky disease	Absent	34/300	86.2 (2.4)	0.720	5/300	97.4 (1.2)	**0.019**
	Present	10/79	85.3 (4.4)		5/79	91.1 (3.8)	
Extranodal involvement, n (%)	Absent	27/267	87.8 (2.3)	0.128	5/267	97.4 (1.2)	0.122
	Present	17/112	81.7 (4.5)		5/112	92.8 (3.3)	
International prognostic score	0–2	22/267	89.7 (2.1)	0.001	3/267	98.3 (1.0)	**0.003**
	≥3	22/112	77.0 (4.8)		7/112	90.8 (3.5)	
Interim PET-CT Deauville score	1 or 2	31/302	87.6 (2.3)	0.087	9/302	95.6 (1.5)	0.440
	3	13/77	79.8 (5.1)		1/77	98.1 (1.8)	
Number of ABVD cycles	2	32/280	85.1 (2.6)	0.202	7/280	96.1 (1.5)	0.692
	3	8/46	83.9 (5.6)		2/46	93.7 (4.3)	
	4	4/53	91.1 (4.3)		1/53	97.6 (2.4)	

Bold text indicates statistical significance at *p* < 0.05 level. *Abbreviations:* ABVD: Adriamycin (doxorubicin), Bleomycin, Vinblastine, and Dacarbazine, ECOG-PS: Eastern Cooperative Oncology Group- Performance Status, OS: Overall Survival, PET-CT: Positron Emission Tomography- Computed Tomography, PFS: Progression-Free Survival, SE: Standard Error.

**Table 4:** Factors associated with response on interim PET-CT in patients receiving 2 cycles of ABVD.

Characteristics	Interim PET-CT Deauville Score		
	Score 1 or 2 (n = 216)	Score 3 (n = 64)	*p* Value
Age at diagnosis, median (range), yr	33 (18–78)	37 (18–71)	0.340
Age at diagnosis, n (%)			
18–24 years	55 (25.5)	15 (23.4)	
25–44 years	94 (43.5)	24 (37.5)	
45–59 years	42 (19.4)	15 (23.4)	0.673
≥60 years	25 (11.6)	10 (15.6)	
Male sex, n (%)	100 (46.3)	39 (60.9)	**0.040**
ECOG-PS, n (%) 0	113 (52.3)	27 (42.2)	0.403
1	85 (39.4)	29 (45.3)	
2	17 (7.9)	8 (12.5)	
3	1 (0.5)	0 (0.0)	
Ann Arbor stage, n (%)			0.587
II	78 (36.1)	26 (40.6)	
III	72 (33.3)	17 (26.6)	
IV	66 (30.6)	21 (32.8)	
B symptoms, n (%)	129 (59.7)	41 (64.1)	0.532
Bulky disease, n (%)	34 (15.7)	11 (17.2)	0.782
Extranodal involvement, n (%)	63 (29.2)	21 (32.8)	0.576
International prognostic score, n (%)			0.06
0–2	161 (74.5)	40 (62.5)	
≥3	55 (25.5)	24 (37.5)	

Bold text indicates statistical significance at *p* < 0.05 level. *Abbreviations:* ECOG-PS: Eastern Cooperative Oncology Group-Performance Status, PET-CT: Positron Emission Tomography-Computed Tomography.

**Table 5:** Relationship between prognostic factors and progression-free survival and overall survival in patients receiving 2 cycles of ABVD.

Characteristics	Groups	Progression-Free Survival			Overall Survival		
		Events/n	3-Year PFS, % (SE)	*p* Value	Events/n	3-Year OS, % (SE)	*p* Value
Age at diagnosis	18–44 yr	20/188	86.7 (2.9)	0.574	1/188	99.2 (0.8)	**0.002**
	≥45 yr	12/92	81.9 (5.5)		6/92	89.4 (4.5)	
Sex	Male	13/139	89.3 (3.2)	0.328	2/139	97.2 (2.1)	0.265
	Female	19/141	81.2 (4.1)		5/141	94.9 (2.3)	
ECOG-PS	0 or 1	27/254	86.3 (2.7)	0.192	4/254	97.2 (1.4)	**0.002**
	2 or 3	5/26	75.6 (9.8)		3/26	85.7 (7.9)	
Ann Arbor stage	II	10/104	87.2 (4.0)	0.328	1/104	98.5 (1.5)	0.198
	III	9/89	87.6 (4.0)		2/89	96.8 (2.3)	
	IV	13/87	79.2 (6.2)		4/87	91.6 (4.6)	
B symptoms	Absent	8/110	88.8 (3.9)	0.143	0/110	100 (0.0)	**0.042**
	Present	24/170	83.0 (3.5)		7/170	93.8 (2.4)	
Bulky disease	Absent	25/235	85.9 (2.9)	0.275	4/235	97.1 (1.5)	**0.050**
	Present	7/45	81.2 (6.6)		3/45	90.9 (5.1)	
Extranodal involvement n (%)	Absent	18/196	87.3 (2.9)	**0.039**	4/196	97.1 (1.5)	0.409
	Present	14/84	79.1 (5.9)		3/84	93.3 (4.1)	
International prognostic score	0–2	16/201	89.2 (2.7)	0.002	2/201	98.5 (1.1)	**0.009**
	≥3	16/79	74.5 (6.4))		5/79	90.0 (4.6)	
Interim PET-CT Deauville score	1 or 2	21/216	86.9 (3.0)	0.102	7/216	94.8 (2.0)	0.136
	3	11/64	79.1 (5.7)		0/64	100 (0.0)	

Bold text indicates statistical significance at *p* < 0.05 level. *Abbreviations:* ECOG-PS: Eastern Cooperative Oncology Group-Performance Status, OS: Overall Survival, PET-CT: Positron Emission Tomography-Computed Tomography, PFS: Progression-Free Survival, SE: Standard Error.

## Data Availability

All data that supports the findings of this study are available within the article and from the corresponding author.

## References

[1] Shanbhag S., Ambinder R.F. (2018). Hodgkin Lymphoma: A Review and Update on Recent Progress. CA Cancer J. Clin..

[2] Borchmann P., Ferdinandus J., Schneider G., Moccia A., Greil R., Hertzberg M., Schaub V., Hüttmann A., Keil F., Dierlamm J. (2024). Assessing the Efficacy and Tolerability of PET-Guided BrECADD versus eBEACOPP in Advanced-Stage, Classical Hodgkin Lymphoma (HD21): A Randomised, Multicentre, Parallel, Open-Label, Phase 3 Trial. Lancet.

[3] Herrera A.F., LeBlanc M., Castellino S.M., Li H., Rutherford S.C., Evens A., Davison K., Punnett A., Parsons S.K., Ahmed S. (2024). Nivolumab+AVD in Advanced-Stage Classic Hodgkin’s Lymphoma. N. Engl. J. Med..

[4] Brittain D., Akhtar S., Rodrigues S., Patel M., Moodley D., Singh J.P., Dreosti L.M., Mohamed Z., Al-Mansour M., Alzahrani M. (2024). Treatment Patterns and Clinical Outcomes in Patients with Hodgkin Lymphoma from Saudi Arabia, Türkiye, and South Africa: Subgroup Analysis from the International Multicenter Retrospective B-HOLISTIC Study. Turk. J. Haematol..

[5] Johnson P., Federico M., Kirkwood A., Fosså A., Berkahn L., Carella A., d’Amore F., Enblad G., Franceschetto A., Fulham M. (2016). Adapted Treatment Guided by Interim PET-CT scan in Advanced Hodgkin’s Lymphoma. N. Engl. J. Med..

[6] Luminari S., Fossa A., Trotman J., Molin D., d’Amore F., Enblad G., Berkahn L., Barrington S.F., Radford J., Federico M. (2024). Long-Term Follow-Up of the Response-Adjusted Therapy for Advanced Hodgkin Lymphoma Trial. J. Clin. Oncol..

[7] Barrington S.F., Kirkwood A.A., Franceschetto A., Fulham M.J., Roberts T.H., Almquist H., Brun E., Hjorthaug K., Viney Z.N., Pike L.C. (2016). PET-CT for Staging and Early Response: Results from the Response-Adapted Therapy in Advanced Hodgkin Lymphoma Study. Blood.

[8] Iftikhar I., Abbas M., Afzal M., Bilal H., Uzair M., Qasim M., Ahsan B., Ahmad U., Bokhari S.W.I. (2025). Real-World Outcomes of PET-Adapted Treatment for Classic Hodgkin’s Lymphoma: A Study from a Single Tertiary Care Center. Cureus.

